# What Would Have Helped People With Profound Intellectual and Multiple Disabilities in the UK During COVID‐19?

**DOI:** 10.1111/jppi.70000

**Published:** 2024-11-21

**Authors:** Jill Bradshaw, Roseann Maguire, Amanda Gillooly, Christopher Hatton, Sue Caton, Andrew Jahoda, Edward Oloidi, Laurence Taggart, Stuart Todd, Richard Hastings

**Affiliations:** ^1^ Tizard Centre University of Kent Kent UK; ^2^ Institute of Health and Wellbeing University of Glasgow Glasgow UK; ^3^ Department of Social Care and Social Work Manchester Metropolitan University Manchester UK; ^4^ Unit for Development in Intellectual and Developmental Disabilities University of South Wales Cardiff UK; ^5^ Institute of Nursing and Health Research University of Ulster Belfast UK; ^6^ Centre for Research in Intellectual and Developmental Disabilities University of Warwick Coventry UK

**Keywords:** COVID‐19, family carers, mental health, physical health, profound intellectual and multiple disabilities

## Abstract

People with profound intellectual and multiple disabilities can be excluded from research and relatively little is known about the experiences of people with profound intellectual and multiple disabilities and their carers during COVID‐19. This paper aims to further explore the impact on this group via information provided by paid and family carers. It focuses on key areas such as access to social and health services in addition to questions about health and well‐being. In contextualising these results, some comparisons are made to impacts on other groups. This paper also explores what we might do better in future to support this population. Carers were invited to complete an online survey about their experiences and the experiences of people they supported during COVID‐19 and to suggest what might have made life better. They were invited to complete this survey at four time points (waves) between December 2020 and December 2022. This paper reports on Waves 1–3, that is to August 2022. Services for people with profound intellectual and multiple disabilities reduced during COVID‐19 and have yet to return to pre‐pandemic levels. People with profound intellectual and multiple disabilities were reported to experience increased social isolation, deteriorating mental and physical health, increased behavioural signs of distress and reduced life skills. Three areas were identified regarding what would have made life better: opportunities for social contact and activities; improved access to health and social care services, and; consistent and responsive staff. Results are explored in the context of current challenges in service provision, including staff retention and shifts in staff culture during the pandemic.

## 1 Introduction

The term ‘profound intellectual and multiple disabilities’ is descriptive rather than diagnostic but is typically understood to include people who have a profound intellectual disability in addition to at least one other disability (e.g., physical or sensory) (Doukas et al. 2017). In their overview of the challenges associated with conducting research on and with people with profound intellectual and multiple disabilities, Maes et al. (2021) noted that research with this group typically involves small numbers of participants. People with profound intellectual and multiple disabilities are a relatively small and heterogeneous group and, therefore, findings from studies containing few participants may not always be easy to generalise.

Studies in the United Kingdom have found that people with intellectual disabilities have been particularly adversely impacted by the social, physical and mental impact of COVID‐19 (Courtenay and Perera 2020) having had more infections and worse outcomes following infections, including mortality, compared with the general population (Henderson et al. 2022; Office for National Statistics 2021). There have been relatively few studies which have focused on the experiences of people with profound intellectual and multiple disabilities during the COVID‐19 pandemic, though the importance of making the needs of these families visible has been highlighted (den Boer, Voermans, and Embregts 2022). den Boer, Voermans, and Embregts (2022) included three case studies from the Netherlands exploring mothers' reflections about the need to balance protecting their children from infection with the potential impact of this (typically fewer activities and social meetings) on their child's quality of life. They also highlighted the negative impact of reduced contact with healthcare professionals on their child. Linden et al. (2022) noted that many families in the United Kingdom had turned to non‐governmental agencies for support as a consequence of this reduction in access to healthcare support. Through focus groups with 24 staff working for these agencies, they highlighted the negative impact of the pandemic on family carers' mental and emotional health, the erosion of trust in statutory services and the need for increased carer support. A publication from this current study, (Caton et al. 2024) found that around half of people with profound intellectual and multiple disabilities had participated in digital activities during COVID‐19 and that for some, carers reported that online activities were something that people would continue to use beyond the pandemic.

### 1.1 The Overall Study

This current project reports data from a UK collaboration as a part of which a large sample of family carers and paid support staff working with this group were recruited and retained throughout the study. This reflects the commitment and support of our partner organisations and advisory groups, who helped shape the research questions and recruitment strategies. Our engagement with our partner organisations and advisory groups included Learning Disability England; PMLD Link; Scottish Commission for Learning Disability; Promoting A More Inclusive Society (PAMIS); Learning Disability Wales; All Wales Forum of Parents and Carers of People with Learning Disabilities; Mencap Northern Ireland; Positive Futures, and; CAN Northern Ireland. We met at regular intervals and shared our emerging findings. The key issues around the impact of COVID‐19 were identified through these meetings, written up as brief project reports and published following feedback from the various organisations who had been involved (Maguire et al. 2020; 2021a; 2021b). Key concerns from these meetings are described below. This process enabled the team to understand (and respond to) emerging issues during the research process. The reports were published to inform policymakers about emerging issues, but the information included was not gathered as a part of a research process. The topics identified as emerging issues were also used to inform topics/question domains that needed to be included as part of the research surveys at each data collection wave.

For the {Coronavirus and People with Learning Disabilities study}, data were collected about experiences during the COVID‐19 pandemic from two cohorts of participants. Cohort 1 was a group of adults who had intellectual disabilities and who were able to take part in an interview with a researcher. Cohort 2 was a group of family carers and support staff for adults with intellectual disabilities, where the person with intellectual disabilities was unable to take part in an interview themselves. Cohort two included family carers and staff supporting someone with profound intellectual and multiple disabilities.

### 1.2 Context of Developments, Measures and Restrictions During the Research Period. What Were the Emerging Issues?

Broadly speaking, the United Kingdom entered lockdown in the second half of March 2020. This meant that people were asked to stay at home, apart from essential activities such as shopping for food. People were also allowed to leave the house for a short period once a day to exercise. People who were considered to be at high risk from COVID‐19 were asked to ‘shield’. Shielding meant that people were asked to take additional precautions to minimise the risk of catching COVID‐19. These additional precautions included not leaving the house to shop or for leisure purposes. Restrictions started to ease in June of 2020, with schools and non‐essential shops opening. Some local lockdowns came into force in July, in response to local levels of COVID‐19. For those areas not experiencing local lockdowns, throughout July and August 2020, there was a further easing of restrictions, with more social activities opening up. By September 2020, at least some restrictions returned, for example, placing limits on the number of people meeting.

Early in the pandemic (Maguire et al. 2020), the reduction or removal of support was identified as a key concern for family carers and paid support staff of people with profound intellectual and multiple disabilities, with reports that some families were continuing to pay for support that they were no longer receiving. For some families, the reduction in support had resulted in them no longer being able to support their family members at home. For other families, reduced support led to increased caring responsibilities. The reduction in services and activities was reported as having a negative impact on the physical and mental health of people with profound intellectual and multiple disabilities, as people had little to do. This issue was exacerbated by the difficulties in being able to access healthcare and the move away from face‐to‐face to remote consultations with health professionals. It was reported that physical examinations were of particular importance in monitoring the health of this group.

Family carers and paid supporters of people with profound intellectual and multiple disabilities reported having very limited involvement in decision‐making around any emergency planning or risk assessment. Keeping in contact when family members lived away from home was identified as a key issue. Restrictions on being able to visit face‐to‐face were a cause of anxiety, particularly when communication between carers and people with profound intellectual and multiple disabilities necessitates close proximity. Carers were very concerned about what might happen should the person they were caring for have to go into hospital.

These reported experiences from project meetings with partner organisations were used to inform the first research survey.

From the end of October 2020 to February/March 2021, there were various short and longer lockdowns and restrictions at slightly different times in the four UK nations.

By January 2021 (Maguire et al. 2021a), family carers of people with profound intellectual and multiple disabilities were increasingly concerned about their relatives becoming withdrawn due to reductions in social contact. They were concerned about the mental health of this group and cognisant that mental health issues might not be recognised in this group and that even if mental health issues were identified, there was very little on offer in terms of support for this. Family carers reported that the closure of services had often meant that their relative also lacked access to health professionals, including therapists. Families often lacked access to appropriate equipment needed to support good physical health and were concerned about the ongoing impact on health. Although many people were accessing groups and services online, access for people with profound intellectual and multiple disabilities was perhaps less usual.

These reported experiences from project meetings with partner organisations were used to inform the second research survey.

Restrictions started to be lifted again in March 2021. By May 2021 (Maguire et al. 2021b), while some services for people with intellectual disabilities were returning, carers of people with profound intellectual and multiple disabilities were balancing the need to shield people from the risk of COVID‐19 with the risks from ongoing social and physical isolation. Carers recognised that people with profound intellectual and multiple disabilities had lost confidence, communication and life skills and feared that some people might never regain their pre‐pandemic skill levels. The negative impact on family carer's physical and mental health was more apparent and the financial impact of caring for someone with profound intellectual and multiple disabilities also became evident.

These reported experiences from project meetings with partner organisations were used to inform the third research survey.

From our initial engagement in project meetings with partners, and from the small amount of existing research internationally, it is clear that people with profound intellectual and multiple disabilities and those caring for them have been disproportionately impacted by the COVID‐19 pandemic. This paper aims to further explore the impact on this group via information provided by paid and family carers through research surveys. It focuses on key areas such as access to social and health services in addition to questions about health and well‐being. In contextualising these results, some comparisons are made to impacts on other groups. This paper also explores what we might do better in future to support this population, which is relevant to all those supporting and caring for people with profound intellectual and multiple disabilities, including staff and services more generally. Consideration of what we might need to do better is partly in relation to things services need to consider if there was a new pandemic but also relevant in terms of the changes that have happened for people with PIMD and the services they access, partly due to the impact of the pandemic.

## 2 Methodology

### 2.1 Procedure

At the start of the survey, Cohort 2 participants (Cohort 2 involved collecting online surveys from family or paid carers of adults with intellectual disabilities with greater support needs) were asked to indicate whether the term ‘profound intellectual and multiple disabilities’ applied to the person they care for. Carers were invited to complete an online survey at three time points. Data were collected from December 2020 to February 2021 (Wave 1), April–May 2021 (Wave 2) and July—August 2021 (Wave 3). These waves refer to our data collection time points and not to waves or peaks of COVID‐19. Responses were entered directly into Qualtrics. The survey questions were developed through structured consultations with advisory groups of people with intellectual disabilities and their families in the four nations of the United Kingdom. We worked directly with organisations that supported people with profound intellectual and multiple disabilities and their families to ensure the questions captured issues of importance. At each wave, draft questions were reviewed by our advisory groups and revised where appropriate. This consultation involved a number of steps, including asking family carers from across the United Kingdom to tell us what the most important issues were and using these to draft survey questions, which were shared with advisory groups in each country for feedback. We did this for each data collection wave. Recruitment of participants was facilitated through our collaborating organisations in each of the four nations (including organisations run by and for families of individuals with profound intellectual and multiple disabilities), social media and wider networks of intellectual disability and carer organisations across the United Kingdom. Potential participants were directed to the online survey via e‐mail, social media or via the research project website. Research ethics approval was obtained from the Manchester Metropolitan University Faculty of Health, Psychology and Social Care Faculty Research Ethics Committee.

### 2.2 Measures

A survey was designed to collect information from carers about the lives of people with intellectual disabilities during the COVID‐19 pandemic. Survey questions were agreed through consultations with stakeholders (groups of people with intellectual disabilities and family carer organisations). The survey included questions about the person's physical health, mental health, service provision, contact with health professions and social lives. Answer categories varied according to the question being asked but were typically positive, negative and do not know, with some additional specific details, for example, ‘good, ok, not very good, not good, don't know’ (in relation to health today) or ‘worse, about the same, better’ (in relation to changes in physical health since a particular time point), or a list of options to select (e.g., in relation to which professionals people had contact with). Full details of questions asked in each wave can be found in the project reports from each wave (Flynn et al. 2021).

At each wave, there were also a relatively small number of open‐ended questions that family and paid carers were invited to complete about the person with learning disabilities that they were supporting and/or caring for. The following open‐ended questions (completed by carers who indicated that the term ‘profound multiple and intellectual disabilities’ applied to the person they were caring for) were analysed and reported in the present paper. Responses to each question ranged from 1 to 14 sentences. Questions differed across time points according to what the current and emerging issues were. For this paper, the following open‐ended questions (in addition to the quantitative data) were included in the analysis:

1. What has life been like for the person you care for/support during the pandemic? (Wave 1 [*N* = 140] and Wave 2 [*N* = 90]).
2. What has been the impact of reduced services and support for the person you care for/support during the pandemic? (Wave 2 [*N* = 68]).
3. If you wish, please describe the impact of visitor restrictions on the person you support/care for. (Wave 1 [*N* = 123], Wave 2 [*N* = 67] and Wave 3 [*N* = 24]).
4. What services does the person you support need that they are not getting at the moment? (Wave 2 [*N* = 76]).
5. What would make the life of the person you support better right now? (Wave 1 [*N* = 140] and Wave 2 [*N* = 91]).
*N* indicates the number of participants responding at each time point. Typically, fewer people completed the survey at the second and third time points and not all respondents chose to answer all of the open‐ended questions.

### 2.3 Analysis

Descriptive statistics (frequencies) are reported for quantitative data. We used generic thematic analysis (Percy, Kostere, and Kostere 2015) to analyse responses to the open‐ended survey questions. Although there were some shorter responses, many answers contained more detail, meaning that the analysis carried out was justified. Generic qualitative analysis is particularly useful in survey research that includes qualitative elements in a mixed design (Percy, Kostere, and Kostere 2015), particularly in large data sets. We selected thematic analysis as we were more interested in using the data to qualify and further understand the views of participants. A content analysis reporting a summary of the answers may have focused more on frequency and less on the context (Vaismoradi, Turunen, and Bondas 2013).

To establish reliability, authors two and three independently reviewed the responses to develop an initial thematic coding framework for each question using NVIVO. Frameworks were compared until an agreement on a final list of codes was reached. We ensured there was a Kappa coefficient of at least 0.61 between raters for each code, which is considered to be substantial agreement (Landis and Koch 1977), by discussing any differences and making minor adjustments to the coding approach. The codes were then organised into thematic clusters, as suggested by Patton (2002). Discussion of the thematic clusters took place between the first three authors. Codes were then reviewed and collapsed into final themes.

## 3 Results

### 3.1 Participants Demographics

One hundred sixty‐six family members or paid carers of people with profound intellectual and multiple disabilities participated at Wave 1 of the study. Carers were family members (87%) or paid carers/support workers (13%). The people with profound intellectual and multiple disabilities they were reporting were aged 16–69 years (*M*: 30.6, SD: 11.9; 81 M, 80 F). Most people with profound intellectual and multiple disabilities were living with family (59%). Others were living with other people with intellectual disabilities (25%) or living alone (9%). People with profound intellectual and multiple disabilities lived in Scotland (47%), England (29%), Wales (12%) and Northern Ireland (12%).

### 3.2 Quantitative Findings

Prior to the pandemic, 68% of people with profound intellectual and multiple disabilities in the study were reported to have regularly used day services. These services, along with respite services and community activities had reduced or ceased for 97% of people at the first time point of the study in December 2020. By the third time point (August 2021), 63% of people who had attended day service pre‐pandemic had returned to some form of day service provision, 12% of people had returned to respite services and 36% of people were attending community activities. In August 2021, more than half of carers (52%) reported that people with profound intellectual and multiple disabilities were receiving less support and almost half (47%) reported that they were paying for services that they were not receiving.

At the third time point (August 2021), 50% of people who did not live with family had some form of restrictions imposed on visits from family, friends and professionals and 5% of people had still not seen their family and friends in person. At this point, around half of carers (55%) felt that visitor restrictions were still having a negative impact on the person they supported and almost a quarter of people (22%) with profound intellectual and multiple disabilities were still shielding. People with profound intellectual and multiple disabilities were less likely to be keeping in touch with family and friends online.

Around 60% of people had contact with health professionals at the third time point. Most GP consultations (61%) were by telephone, 6% by video and a third were in person. In contrast, most community nurse (58%) and allied health professional (72%) consultations were in person, by telephone (31% and 18%, respectively) and by video (11% and 9%, respectively). Almost half (46%) of all consultations with psychiatrists, psychologists or counsellors were by telephone, 36% were in person and 18% by video.

At the third time point (August 2021) only 29% of people had received their annual health check in 2021. One‐fifth of people had a medical test, operation or hospital appointment cancelled in the previous 4 weeks and 44% had been waiting at least 6 months for a medical test, operation or hospital appointment. Almost a third of people (30%) had reported a new or worsening health condition in the last 4 weeks.

The majority of people with profound intellectual and multiple disabilities were reported to be feeling worried or anxious at least some of the time at the second and third time points (80% and 69%, respectively), sad or down (74% and 68%, respectively), and angry or frustrated (79% and 80%, respectively). Most people (54%) were receiving support from family for these feelings, with only 5% receiving support from a mental health professional.

### 3.3 Qualitative Findings

Responses to the open questions generated five overlapping themes. These themes were: (1) increased social isolation, (2) concerns about mental health, (3) concerns about physical health, (4) increased behavioural signs of distress and (5) reduced life skills.

1. Increased social isolation

People with profound intellectual and multiple disabilities were reported to have experienced increased isolation during the pandemic, whether they lived in a residential setting or at home:

She's very isolated even within the home she lives in, cut off from family, activities ceased outside home, most external people not able to attend home.

Isolating from rest of family and friends other than main carer. Isolated at home after discharge as no support workers and no community activities.

Carers' responses pointed to a direct link between the impact of general restrictions and the reduction or withdrawal of support services and people's increased sense of social isolation:

Life has been very hard for her as she is stimulated by interaction with people and unfortunately due to day centre still being closed and restrictions set by the government, she has not been able to see people.

Life has been extremely difficult for our daughter as she has been confined to the house 90% of the time. Her disability is very profound and she does not understand toys/objects and therefore her stimulation comes from interaction with other people and due to the closure of her day centre and the lockdown this has not been able to happen.

Their responses emphasised the importance of routine for many people with PIMD:

It has been stressful, my daughter very much thrives on routine and sensory activities, this has been taken away as her day centre has been closed.

Carers highlighted the significance of social contact for the well‐being of people with PIMD as illustrated by the following insight from a family carer:

I believe that she also misses her ‘friends’ and contemporaries that she used to see on a regular basis before March 2020, I think that contact is very important for people with PIMD. We may not understand the relationships they have but they are very important to the individual and I can see how important it is for my daughter if by chance we meet a person she knows out and about or if we're lucky enough to share a session with one of her friends, they are both delighted to see each other and the joy it brings is incredible.

2 Concerns about mental health

Carers of people with profound intellectual and multiple disabilities reported a deterioration in the mental health of the person they support. Some carers talked about specific mental health issues, for example, depression.

You can physically see her closing down and she appears very depressed and it is now difficult to get any response from her.

She had to go on antidepressants to stop her from seriously self‐harming.

Others commented on a change in the person they support, including a loss of interest or change in character:

He is not the person he was in March—he was generally a happy young man who enjoyed life but now he sits in his wheelchair or stands in his standing frame or walker with his head hanging down, he doesn't engage in anything and it is hard to keep him entertained all day. He generally seems disinterested in anything whereas before he was aware of everything going on around him, interacted well with others and had an inquisitive/mischievous nature.

She uses touch to communicate as she cannot form words, so not being able to see and touch the ones she loves has been so difficult and upsetting… She gets very frustrated and cries a lot, especially when on the phone to myself and my family asking when we are going to pick her up. I think her heart is breaking not seeing us, I know mine is.

Carers' responses indicated a direct link between the loss of routine, service closure and deteriorating mental health:

He suddenly had all his contact with the outside world stopped. We've tried to explain the big bad sick to him, he's very anxious and worried.

Adam's (pseudonym) mental health suffering that everything had changed he wasn't seeing friends or going out with the daycare group.

As time went on with no day centre and only using a support worker to let me go out and shop, he has become more lethargic. He is sleeping more in the morning, has been eating more, weight has gone up.

Some carers noted improvements in the person's mental health following contact with their loved ones, further highlighting the association between social isolation and deteriorating mental health:

He sometimes has sleep problems that are resolved when one of us manages a visit.

3 Concerns about physical health

Carers reported a deterioration in the physical health of the person with profound intellectual and multiple disabilities. This was linked to the withdrawal or reduction of allied health professional services and other medical interventions:

Body shape terrible as no physio.

Restricted and has made her physical health worse with lack of hydrotherapy, music therapy etc eating more, has put on weight.

She have gone downhill generally, More seizures, more Gastric and Bowel problems most likely attributed to less focus on health care from professionals due to restrictions in movement and surgery opening, simply harder to get attention when required.

He has not been able to access any of the usual medical reviews and his health has suffered as a result.

Carers also reported a link between deteriorating physical health and a lack of stimulation:

Is epileptic, which is well controlled but since lockdown she has had more seizure activity which I believe is due to the lack of stimulation.

4 Increased behavioural signs of distress

Carers of people with profound intellectual and multiple disabilities reported an increase in behavioural signs of distress:

A severe decline in behaviour. Frequently walking around shouting and rocking. Sleep patterns completely disrupted and frequent bouts of bad temper and frustration. An almost complete unwillingness to cooperate with carers at home.

In common with the other identified themes, carers pointed to the reduction in service provision and support as key factors contributing to increased behavioural difficulties:

His behaviour at times is very challenging and I'm sure some of it is boredom with no day centre.

My daughter has no way of understanding what is happening. Her response to loss of meaningful activities has been to attempt control via refusal… All of her team have noticed, more refusals, more chronic constipation, more manic outbursts, more sleeplessness, more lashing out, even a return of head butting, although thankfully she is still eating well, so no return of her anorexia. She is napping much more, and really missing her regular weekly massage. This has eased her physical and mental so well, it is understandable she is now very tense much of the time. Has developed a new teeth clenching behaviour, which results in great tension.

Carers' responses not only revealed their understanding of the reasons for an increase in behavioural difficulties but highlighted the importance of meaningful activities and social interaction for the positive health and well‐being of people with profound intellectual and multiple disabilities.

5 Reduced life skills of people with PIMD

Carers reported a loss or reduction of life skills. Some carers believed this to be a consequence of deteriorating physical health:

His epilepsy is worse, and this has affected what little mobility he had. He is no longer able to crawl and won't take steps in his walker.

While others highlighted the impact shielding had on their relative's mobility:

Shielding, particularly first time round when she wasn't allowed out at all has impacted her mobility hugely. We bought her a floor cycle to keep strength but her confidence to walk outside has gone. Her fall risk has increased markedly.

Carers also highlighted the impact the pandemic had on their relative's confidence and social skills:

He's definitely slipped back in terms of social skills and life skills.

She is less confident in her interactions with others.

Carers raised concerns that this deterioration of life skills may have long‐term consequences.

### 3.4 What Would Have Helped People With PIMD During the Pandemic?

The evidence from the analysis of carers' responses to what life was like for people with profound intellectual and multiple disabilities has highlighted the detrimental impact reduction in support services, reduced access to health services, including allied health care professionals, and reduced social contact and meaningful activities have had on people's health and well‐being. Therefore, it is not surprising that carers' responses to what would make life better generated broad themes around these categories. Three themes were identified: (1) opportunities for social contact and activities, (2) provision of and better access to health and social care services and (3) consistent and responsive staff.

1. Opportunities for social contact and activities

Carers reported that people with profound intellectual and multiple disabilities' lives would have been improved by opportunities for social contact with family and friends, both at home and in the community:

Seeing and having meaningful time (close, personal contact) with the significant people in his life—family & friends.

Getting out to do her activities and having people call to our house.

Carers also stated that opportunities to participate in community activities would have helped people during the pandemic. While some carers specified activities, for example, ‘cycling, swimming, sailing, Boccia’. Other responses were more general, for example, ‘access to community activities’.

2 Provision of and better access to health and social care services

Carers reported that the re‐opening of day and respite services would make life better for people and suggested that this could be feasible with increased funding:

Open up the day centres with increased funding to allow social distancing that might be necessary.

In addition to better access to health services for people with profound intellectual and multiple disabilities ‘better access to services such as dental treatment, community nursing’, carers also highlighted the importance of contact with allied health professional services ‘physiotherapy, hydrotherapy, rebound therapy’.

3 Consistent and responsive staff

Carers wanted staff who were trained to support people with profound intellectual and multiple disabilities:

Staff understanding guidelines re support to a vulnerable person.

They suggested that ‘a consistent staff team who can actually work as a team’ would be beneficial and were looking for ‘staff to be a bit more imaginative regarding the things they can do in the home to stimulate and entertain the person’.

## 4 Discussion

### 4.1 The Experiences of People With and Without Profound Intellectual and Multiple Disabilities

People with profound intellectual and multiple disabilities in this study experienced negative impacts from the pandemic. This was particularly around social isolation, poorer mental and physical health, reduced life skills and increased behavioural difficulties. The first three of these may not be dissimilar from the impact on people without profound intellectual and multiple disabilities, and the impact is similar to that experienced by people with intellectual disabilities generally (Brooks et al. 2020). However, there are some important differences.

Life was lonelier for most people during the pandemic (Pai and Vella 2021) and many people with intellectual disabilities living in adult social care were living under visitor restrictions which contributed to social isolation (Araten‐Bergman and Shpigelman 2021; Honingh et al. 2022), for many people, social contact outside of their immediate household continued online (Caton et al. 2024). Similarly, while most people's activities were reduced (Caputo and Reichert 2020) and many people with intellectual disabilities experienced reduced services (Flynn et al. 2021), for most people with intellectual disabilities, opportunities to join activities were still provided, all be‐it remotely.

Caton et al. (2023) found that people with profound intellectual and multiple disabilities had less access to the internet at home during the pandemic than people with mild or moderate intellectual disabilities, a difference that might be partly explained by perceptions that people with profound intellectual and multiple disabilities would not be able to participate in online events. Digital participation was successful for some people with profound intellectual and multiple disabilities, particularly where communication partners were able to develop skills around including this group (Kversøy et al. 2022). Therefore, it seems likely that there may have been missed opportunities to involve people with profound intellectual and multiple disabilities digitally.

People with intellectual disabilities experience poorer mental health during the pandemic (Lunsky et al. 2022). This was also the case for people with profound intellectual and multiple disabilities (Flynn et al. 2021). What differs for people with profound intellectual and multiple disabilities is that poorer mental health is harder to recognise (Sheehy and Nind 2005), and the risk of missing mental health issues may have been exacerbated given reduced contact with health professionals during the pandemic (Flynn et al. 2021).

Lack of, or a reduction in, activity is also a particular issue for this group. People with profound intellectual and multiple disabilities may find it harder to occupy themselves (Mansell and Beadle‐Brown 2012). They may also require specialist support and equipment to support their physical health. As noted previously, contact with health professionals was reduced and many families did not have access to specialist support or equipment. Whereas for the majority of people, with time and intervention, fitness, skills and confidence are likely to return, carers were concerned that people with profound intellectual and multiple disabilities may never regain lost skills. Finally, it is important to note that poorer mental health, reduced activities and social isolation may contribute to behaviours that challenge.

### 4.2 What Do We Need to Consider in a Future Pandemic?

In future, making sure that people have digital access and providing training around supporting online interactions might be one way in which people with profound intellectual and multiple disabilities might learn skills and be able to maintain social interactions and reduce loneliness. There is a need to offer both face‐to‐face and online consultations so that people with profound intellectual and multiple disabilities can be supported to engage with health professionals in a way that best suits them on each occasion. Although connection and activities may, with skilled and creative communication partners, continue online for at least some people, finding ways to continue with physical activity should be a priority. Protecting existing skills is of vital importance for people with profound intellectual and multiple disabilities and health and social care professionals should work with carers to think about how physical activities might be included in daily activities, without the need for specialist equipment and without increasing carer workload and responsibilities as far as possible. The retention of health and social care staff needs to be prioritised so that skilled and knowledgeable staff continue to work with people with profound intellectual and multiple disabilities and their families to enable them to have a good quality of life.

### 4.3 What Else Do We Need to Consider for This Group of People?

By the end of March 2022, all legal restrictions were removed. This included removing the legal requirement to self‐isolate following a positive test for COVID‐19 and the requirement to wear a facemask. By June 2022 (Coronavirus and Learning Disabilities Study Team 2022), services had still not returned to the levels, types and nature of support that they were at pre‐pandemic. Concerns were raised about the impact of staff recruitment and retention issues on the quality of support provided and that staff who were unfamiliar with their relative may be even less likely to be able to support their relative to regain skills that had been lost during the pandemic. Families also noted a shift in culture during the pandemic, where the focus of support had moved from rights to protection and were anxious about whether this might become the new normal. Impact on physical health in particular through reduced contact with allied health professionals, such as physiotherapists, was an ongoing issue and many routine health checks (such as the dentist) were still not available for this group. Some providers were seen as risk averse and to be continuing to impose restrictions without balancing the risks of COVID‐19 and the risks to physical and mental health from ongoing isolation. With the cost of living crisis now in evidence, it was noted that the impact on families caring for someone with profound intellectual and multiple disabilities was likely to increase.

### 4.4 Limitations

This paper reports data from a larger study. This study aimed to respond to emerging issues for people with profound intellectual and multiple disabilities as reported by carers and organisations by gathering data across three time points. The focus on reported issues may have meant that opportunities to include topics from a broader perspective were missed.

Our analysis relied on paid and family carer‐reported data about someone with profound intellectual and multiple disabilities rather than on data from the person themselves (e.g., via observation) and was, therefore, from one perspective only. However, people with profound and multiple learning disabilities who are unable to communicate their own views, rely on those they are closest to and know them best, to ensure that their lives and experiences are included in research (Rushton, Kossyvaki, and Terlektsi 2023).

Although it is possible that respondents may have differed in their interpretations of the term profound intellectual and multiple disabilities, families and paid carers were seen as the experts in deciding whether this term applied to the person they were completing the survey about (Caton et al. 2023). It is also noteworthy, in this regard, that participants were recruited with the support of organisations by and for the families of individuals with profound intellectual and multiple disabilities.

The data were collected via written responses to surveys over four time points. This paper wholly focuses on the first three time points. Written responses were helpful in that they enabled participants to provide information about each area but did not provide an opportunity for researchers to clarify understandings or to further probe issues raised.

## Ethics Statement

Research ethics approval was sought and obtained from the Faculty of Health and Education Research Ethics Committee at Manchester Metropolitan University.

## Conflicts of Interest

The authors declare no conflicts of interest.

## Data Availability

The data that support the findings of this study are available from the corresponding author on reasonable request.
